# Assessment of Thrombophilic Abnormalities During the Active State of Inflammatory Bowel Disease

**DOI:** 10.4103/1319-3767.41743

**Published:** 2008-10

**Authors:** Maha M. Maher, Somaya H. Soloma

**Affiliations:** Departments of Internal Medicine and Clinical Pathology, Mansoura and Al-Azhar University, Cairo, Egypt

**Keywords:** Active state, inflammatory bowel disease, thrombophilic markers

## Abstract

**Background/Aims:**

Thromboembolic disease has been recognized as a complication of inflammatory bowel disease (IBD). The relative contributions of inherited or acquired thrombophilia and the inflammatory response to the mechanism of this tendency are unclear. Thrombotic events are more common in active disease although significant numbers also occur spontaneously. The aim of this study was to investigate common thrombophilic markers in patients with active IBD.

**Methods:**

Twenty-six patients with IBD who had active disease, and 40 sex- and age-matched non-IBD patients were recruited into the study. For all the subjects, complete blood counts, C-reactive protein levels, erythrocyte sedimentation rate, International normalized ratio, activated partial thromboplastin time, and levels of lupus anticoagulant, anticardiolipin antibodies (ACA IgG), proteins C and S, antithrombin-III (AT-III), and factor V were measured.

**Results:**

The International normalized ratio, activated partial thromboplastin time, and levels of proteins C and S were comparable between the two groups. However, antithrombin-III levels were significantly lower in the IBD group as compared with that in the healthy control group (*P* < 0.001). ACA IgG was detected in one patient in the IBD group. Factor V Leiden mutation was present in 3.8% of the patients in the IBD group, whereas the prevalence was 2.5% in the control group. Significantly elevated platelet counts were observed in patients with active Crohn's disease compared with that in the control group (*P* < 0.001), but they were not significantly increased in active ulcerative colitis (*P* = 0.231).

**Conclusions:**

The present study failed to establish a strong association between the common thrombophilic markers and the active clinical course of IBD, with the exception of high platelet counts and lower levels of AT-III in the IBD group as compared with those in the control group. All other parameters of thrombophilia were comparable between the two groups.

Crohn's disease (CD) and ulcerative colitis (UC), the two major forms of inflammatory bowel disease (IBD), are chronic inflammatory conditions characterized by local and systemic inflammation. It is well established that genetic predisposition and immune dysregulation play key roles in IBD pathogenesis. However, clinical experience and research have clearly demonstrated that a hypercoagulable state and a prothrombotic condition exist in both forms of IBD, whereas coagulation abnormalities are an intimate part of IBD's clinical picture.[1]

IBD patients frequently experience systemic thromboembolic complications that represent an important cause of morbidity and mortality.[2] A recently performed population-based study has shown that IBD patients have a threefold higher risk of developing deep venous thrombosis and pulmonary embolism when compared with the general population.[3]

Abnormalities of the genes encoding for antithrombin III, protein C, and protein S are rare (found in less than 1% of the population). Their presence has been associated with a high (more than tenfold) risk of venous thromboembolic events (VTE). Factor V Leiden (FVL) genetically inherited mutation is currently recognized as the most common genetic defect associated with thrombophilia. It is present in 4–6% of the general population and is associated with a six and 80fold higher risk of VTE in heterozygote and homozygote individuals, respectively.[4]

The search for common thrombophilic markers in IBD has, however, revealed conflicting results.[5–7] Low levels of natural coagulation inhibitors such as antithrombin III (AT-III) and proteins C and S have been observed in some patients with IBD[8,9] but not in the entire population.[10,11]

In the past few years, much research has focused on the inherited thrombophilic risk factors in IBD as a way to clarify their possible participation in associated macrovascular thrombosis.[12] Two reports disclosed that 45% of CD patients[13] and 4 of 11 patients with thrombosis had inherited the FVL mutation.[14] Therefore, it is still questionable whether the entire IBD patient population should be studied for the presence of common thrombophilic markers.[15,16]

The degree of activity and the extent of inflammatory intestinal disease are generally considered to correlate well (but not invariantly) with the patient's risk for a thromboembolic (TE) event.[17,18]

We have therefore explored the prevalence of such thrombophilic abnormalities in a group of active IBD patients who had no history of thromboembolic disease and compared them with healthy age- and sex-matched controls.

## PATIENTS AND METHODS

### Patients

Twenty-six patients with IBD who had been followed up at King Fahd Hospital, Al Hofuf, Department of Gastroenterology, were recruited into the study. There were seven CD (four men and three women; mean age = 32 years) and 19 UC (eight men and 11 women; mean age = 36 years) patients.

All patients had a definitive diagnosis of UC or CD that had been confirmed by radiological, endoscopic, and histological studies. Endoscopic reevaluation was done at the time of the study for confirmation of the extent of disease for all patients. These were compared with 40 blood donors—healthy controls (HC)—who were matched to the patient population for age and gender.

None of the patients was taking any medications (such as oral anticoagulants, aspirin, nonsteroidal antiinflammatory drugs, and contraceptives) that might have caused platelet or coagulation abnormalities during the last 8 weeks before blood sampling. Impaired renal or liver function, myeloproliferative disorders, and cancer were exclusion criteria. None of the patients in the IBD group had had any previous thrombotic episode.

Disease activity in CD and UC was evaluated by the use of the Crohn's Disease Activity Index (CDAI) score[19] (Appendix 1) and the Truelove-Witts grading system[20] (Appendix 2), respectively..The clinical data of the IBD patients have been summarized in Table 1.

**Appendix 1:** Crohn's disease activity index (CDAI) score

Clinical or laboratory variable	Weighting factor
Number of liquid or soft stools each day for seven days	× 2
Abdominal pain (graded from 0 to 3 on severity) each day for seven days	× 5
General well being, subjectively assessed from 0 (well) to 4 (terrible) each day for seven days	× 7
Presence of complications*	× 20
Taking Lomitil or opiates for diarrhea	× 30
Presence of an abdominal mass (0 as none, 2 as questionable, 5 as definite)	× 10
Absolute deviation of Hematocrit from 47% in men and 42% in women	× 6
Percentage deviation from standard weight	× 1

* One point each is added for each set of complications:
the presence of joint pains (arthralgia) or frank arthritis inflammation of the iris or uveitis presence of erythema nodosum, pyoderma gangrenosum, or aphthous ulcers anal fissuresanal fissures, fistulaeanal fissures, anal fissures, or abscessesanal fissures, or anal fissures, or other fistulae fever (> 100°F) during the previous week Remission of Crohn's disease is defined as a CDAI < 150.

**Appendix 2:** Truelove – Witts grading system

Parameter	Severe	Mild	Remission
Bowel movements	≥ 6 per day	≤ 4 per day	1–2 per day
Blood in stool	Macroscopic	None to small	None
Temperature	Mean evening temperature > 99.5°F (37.5°C), OR a temperature ≥ 100°F (37.8°C) on at least two days out of four	No fever	No fever
Heart rate	≥ 90 per minutes	No tachycardia	No tachycardia
Hemoglobin	≤ 75% of normal, with allowance for recent transfusion	None to mild	Normal or returning to normal
ESR	> 30 mm/h	≤ 30 mm/h	Normal or returning to normal

**Table 1:** Clinical characteristics of the study population data

	Ulcerative colitis (*n* = 19)	Crohn's disease (*n* = 7)	Healthy subjects (*n* = 40)
Sex (male/female)	8/11	4/3	23/17
Age range (mean)	29–47(36)	18–40(32)	21–47(34)
Disease presentation			
Moderate	12	5	-
Severe	7	2	-
Extent of disease			
Left-side colitis	(7)	Lleocecal (5)	-
Pancolitis	(7)	Small intestinal	-
Ulcerative proctitis	(5)	Colonic (2)	-
Treatment			
Salazopyrine	17	7	-
Oral steroids	8	4	-
Azathioprine	1	1	-
Metronidazole	-	3	-
None	-	-	-

Some patients take more than one drug

### Methods

#### Complete blood count

Blood was collected into tubes containing dipotassium edetic acid (EDTA; 1.3 mg/ml) for the determination of a complete blood count. All measurements were performed within 2 h of blood collection because of the known effect of EDTA on platelet volume.[21] All blood samples were analyzed on a Cell-Dyn 3200 system (Abbott Laboratories, Wiesbaden, Germany), which was subject to daily quality control. The range of normal values for platelet counts in the laboratory is 150–450 (×109/L).

#### C-reactive protein and erythrocyte sedimentation rate

Serum samples were obtained and were drawn into 10-ml serum separator tubes and allowed to clot for 30 min before centrifugation at 2000 rpm for 10 min. The serum was removed, and C-reactive protein (CRP) and erythrocyte sedimentation rate (ESR) were determined using automatic devices according to conventional methods.

#### Hemostasis laboratory methods

Venous blood was collected into 0.129 M trisodium citrate (1:10), and the plasma samples were stored at −80°C until the assays for activated partial thromboplastin time, international normalized ratio, PT, protein C,[22] protein S Ac,[23] antithrombin III (A),[24] FVL (ProC Global, Dade Behring , U.S.A.), lupus anticoagulant (LA1 Screening Reagent/LA2 Confirmation Reagent, Dade Behring, USA) anticardiolipin antibody (ACA) IgG and IgM (Dade Behring, USA).

### Statistical analysis

Data are expressed as medians with interquartile ratios (IQR) and mean ± standard deviation (SD). The Mann–Whitney U test was used to compare nonparametric variables. Differences between the qualitative variables in the two groups were analyzed by the chi-square test. A ***P*** value of less than 0.05 was considered to be significant. Statistical Package for Social Sciences (SPSS v 11.0) software was used for statistical analyses.

## RESULTS

All the patients with UC had moderate or severe disease according to the Truelove-Witts scale, and all patients with CD had scores more than 220 according to the CD activity index. The distribution of CD was ileocecal in 71.4% and small intestinal and colonic in 28.6% of the patients. The extent of UC was pancolonic in 36.8%, left-sided in 36.8%, and rectal in 26.3% of the patients.

The platelet counts, ESR, CRP, and WBC counts of patients with IBD and healthy blood donors have been shown in Table 2. Significantly elevated platelet counts (thrombocytosis is defined as a platelet count greater than 450 ×109/L) were observed in patients with active CD compared to the control group ***P*** < 0.001), but they were not significantly increased in active UC (***P*** = 0.231).

**Table 2:** Platelet counts, mean ESR, CRP, and WBC counts in healthy blood donors and patients with IBD

	PLT (×10^9^/L)	ESR (mm/first h)	CRP (mg/dl)	WBC (mm^3^)
Controls	231(22)	18.1	< 0.5	6200
CD	382 (130)*	56.8*	3.84*	10,050
UC	271 (98)	47.3*	2.3*	9320

The values indicate mean ± SD

* *P* < 0.05

The ESR was elevated in 57.1% of the seven CD patients tested and in 52.6% of the 19 UC patients tested. The CRP was elevated in 84.6% of the 26 IBD patients tested.

The International normalized ratio, activated partial thromboplastin time, and protein C and protein S levels were not statistically different (***P*** = 0.624, ***P*** = 0.172, ***P*** = 0.413 and ***P*** = 0.142, respectively) for the two groups. AT-III was significantly lower in the IBD group than in the healthy control group (***P*** < 0.001). Three out of 26 IBD patients have AT-III <70%. The median values of AT-III in the IBD patients and in the control group were 96 and 109.9% respectively (the reference range is approximately 80–130%; Table 3).

**Table 3:** Thrombophilic markers in patients with IBD and the control group data are presented as Median (IQR)

	IBD	Controls	*P*
INR	1.08 (0.16)	1.3 (0.14)	0.624
aPTT	32.6 (7.4)	26.3 (4)	0.172
Protein C	98 (32.71)	97 (39)	0.413
Protein S	83.5 (30.6)	86.2 (20.4)	0.142
AT-III	96 (17)	109.9 (15)	< 0.001

IBD: Inflammatory bowel disease, IQR: Interquartile ratios, INR: International normalized ratio, aPTT: activated partial thromboplastin time, AT III: Antithrombin III

ACA IgGs were detected in one patient in the IBD group but not in the control group. FVL mutation was present in 3.8 and 2.5% of the IBD patients and the control group (one patient was heterozygous for FVL mutation), respectively. The differences for ACA and FVL were not significant (***P*** > 0.05) between the two groups.

## DISCUSSION

Patients with IBD frequently suffer from thromboembolic events, which represent an important cause of morbidity and mortality in these patients.[25] The incidence of systemic thromboembolism (TE) in IBD ranges between 1 and 7.7% in clinical studies,[26] rising to 39–41% in postmortem studies.[1] In IBD, acquired prothrombotic risk factors are frequently observed, such as inflammation, fluid depletion, immobility, surgery, steroid therapy, and the use of central venous catheters.[27] Furthermore, CD has been associated with known risk factors for TE, such as smoking and the use of oral contraceptives.[28] In our population study, we excluded patients with any previous thrombotic events.

In the largest series that has been investigated to date, it seemed that thromboembolic events were more frequent when IBD was in an active phase and were further correlated with the extent of disease (particularly pancolonic involvement in UC patients or colonic involvement in CD patients).[29–31]

All patients included in our study were in an active state of CD evaluated by the use of the Crohn's Disease Activity Index (CDAI) score and in active state of UC evaluated by using Truelove-Witts grading system. Endoscopic reevaluation was done at the time of the study to confirm the extent of the disease.

Despite reports of several qualitative and quantitative abnormalities in hemostatic parameters in IBD patients,[32–35] the reasons for the increased incidence of TE in IBD are nonetheless not completely understood. It seems to be multifactorial, because no consistent unifying etiology has been identified. It has been suggested that at least one prothrombotic risk factor can be detected in most of the thrombotic IBD patients.[36,37] However, other authors[25] have indicated that approximately half of IBD patients develop TE without any identifiable reason, reinforcing the hypothesis that IBD represents a per se risk factor for thrombosis.

It is now well established that platelets behave aberrantly in both CD and UC. An increase in platelet number (“reactive thrombocytosis” defined as a platelet count >450 × 109/L) frequently occurs during the active phase of IBD. The high platelet number correlates well with disease severity.[38] In agreement with the previously mentioned study, our results showed significantly elevated platelet counts in patients with active CD compared with that in the control group, but they were not significantly increased in active UC.

The reason for the greater number of platelets in IBD patients is not well understood, but it is usually considered to be a nonspecific response to inflammation, similar to what occurs in other chronic inflammatory conditions such as rheumatoid arthritis or systemic lupus erythematosus. It has also been proposed that the thrombocytosis of CD and UC could reflect a disturbance in thrombopoiesis.[39]

Deficiencies and functional abnormalities of AT-III and proteins C and S are well-recognized causes of thrombotic disease and account for 14–24% of cases with familial thrombotic disease. The role of well-recognized inherited thrombophilic states, such as deficiencies of plasma AT-III and proteins C and S, as well as resistance to activated protein C is under investigation in IBD patients.[40]

In a previous study,[41] the prevalence of a single prothrombotic abnormality in the IBD group was higher than in the control group (26% and 18%, respectively; ***P*** < 0.02). The prevalence of combined thrombophilic abnormalities in both CD and UC was also higher (22% and 21%, respectively) compared with that of the control group (9%; ***P*** < 0.01). These differences were related to disease activity in CD.[25] Deficiencies of proteins C and S in patients with IBD have been proposed in the studies of Jorens ***et al.***[11] and Aadland ***et al.***[8,9] Other studies, however, did not confirm these data.[4,25]

In the present study, the levels of proteins S and C were not significantly different between the two groups and were both in the normal range in both the IBD and control groups.

The main physiological thrombin inhibitor, AT-III, was found to be significantly reduced in IBD patients in the present study as it was in some other studies.[10,25,42] Our data showed that AT-III levels were significantly lower in the IBD group as compared with the control group. Low circulating AT-III levels may complicate the active disease course of IBD and contribute to the ongoing thrombotic manifestations of IBD.[43]

Published data have mostly shown no differences in the prevalence of FVL in IBD patients with respect to healthy controls.[16,25,44–46] Also considering CD and UC patients separately, only two of 14 studies reported differences in the frequency of FVL carriers.[13,45] In particular, Haslam ***et al.***[45] observed a higher prevalence of FVL in UC but not in CD compared to the controls. On the contrary, Over ***et al.***[13] found a higher frequency in CD than in UC patients or the controls. In the present study, there was no significant link between common prothrombotic mutations and the present active state of IBD as was noted in some previous studies. The presence of antiphospholipid antibodies, a well-known factor of acquired thrombophilia,[47] was detected in one IBD patient in our study.

The present study failed to establish a strong association between the common thrombophilic markers and the active clinical course of IBD, with the exception of high platelet counts and lower levels of AT-III in the IBD group as compared with that in the control group. All other parameters of thrombophilia were comparable between the two groups.

We conclude that patients with IBD do not have a higher prevalence of thrombophilic abnormalities when compared with age- and sex-matched controls, with the exception of high platelet counts and lower levels of AT-III in the IBD patients. However, the number of subjects in this study was small. A much larger study would be desirable to confirm this result, and further studies on platelet function in IBD are recommended.
